# [68Ga]Ga-FAPI-46 PET/CT for Staging Suspected/Confirmed Lung Cancer: Results on the Surgical Cohort Within a Monocentric Prospective Trial

**DOI:** 10.3390/ph17111468

**Published:** 2024-11-01

**Authors:** Lucia Zanoni, Emilia Fortunati, Giulia Cuzzani, Claudio Malizia, Filippo Lodi, Veronica Serena Cabitza, Irene Brusa, Stefano Emiliani, Marta Assenza, Filippo Antonacci, Francesca Giunchi, Alessio Degiovanni, Marco Ferrari, Filippo Natali, Thomas Galasso, Gian Piero Bandelli, Simona Civollani, Piero Candoli, Antonietta D’Errico, Piergiorgio Solli, Stefano Fanti, Cristina Nanni

**Affiliations:** 1 Nuclear Medicine, IRCCS Azienda Ospedaliero—Universitaria di Bologna, 40138 Bologna, Italy; lucia.zanoni@aosp.bo.it (L.Z.); emilia.fortunati@aosp.bo.it (E.F.); claudio.malizia@aosp.bo.it (C.M.); filippo.lodi@aosp.bo.it (F.L.); veronicaserena.cabitza@aosp.bo.it (V.S.C.); irene.brusa@aosp.bo.it (I.B.); stefano.emiliani@aosp.bo.it (S.E.); marta.assenza@aosp.bo.it (M.A.); cristina.nanni@aosp.bo.it (C.N.); 2 Nuclear Medicine, Alma Mater Studiorum University of Bologna, 40138 Bologna, Italy; giulia.cuzzani2@studio.unibo.it; 3 Division of Thoracic Surgery, IRCCS Azienda Ospedaliero—Universitaria di Bologna, 40138 Bologna, Italy; filippo.antonacci@aosp.bo.it (F.A.); piergiorgio.solli@aosp.bo.it (P.S.); 4 Pathology, IRCCS Azienda Ospedaliero—Universitaria di Bologna, 40138 Bologna, Italy; francesca.giunchi@aosp.bo.it (F.G.); alessio.degiovanni@aosp.bo.it (A.D.); antonietta.derrico@aosp.bo.it (A.D.); 5 Interventional Pulmonology Unit, IRCCS Azienda Ospedaliero—Universitaria di Bologna, 40138 Bologna, Italy; marco.ferrari@aosp.bo.it (M.F.); filippo.natali@aosp.bo.it (F.N.); thomas.galasso@aosp.bo.it (T.G.); gianpiero.bandelli@aosp.bo.it (G.P.B.); piero.candoli@aosp.bo.it (P.C.); 6 Department of Medical Physics, IRCCS Azienda Ospedaliero—Universitaria di Bologna, 40138 Bologna, Italy; simona.civollani@aosp.bo.it

**Keywords:** [68Ga]Ga-FAPI, PET/CT, lung cancer, staging

## Abstract

Background/Objectives. To evaluate T&N-staging diagnostic performance of [68Ga]Ga-FAPI-46 PET/CT (FAPI) in a suspected/confirmed lung cancer surgical cohort. Methods: Patients were enrolled in a prospective monocentric trial (EudraCT: 2021-006570-23) to perform FAPI, in addition to conventional-staging-flow-chart (including [18F]F-FDG PET/CT-FDG). For the current purpose, only surgical patients were included. PET-semiquantitative parameters were measured for T&N: SUVmax, target-to-background-ratios (using mediastinal blood pool-MBP, liver-L and pulmonary-parenchyma-P). Visual and semiquantitative T&N PET/CT performances were analysed per patient and per region for both tracers, with surgical histopathology as standard-of-truth. Results: 63 FAPI scans were performed in 64 patients enrolled (26 May 2022–30 November 2023). A total of 50/63 patients underwent surgery and were included. Agreement (%) with histopathological-T&N-StagingAJCC8thEdition was slightly in favour of FAPI (T-66% vs. 58%, N-78% vs. 70%), increasing when T&N dichotomised (T-92% vs. 80%, N-78% vs. 72%). The performance of Visual-Criteria for T-per patient (n = 50) resulted higher FAPI than FDG. For N-per patient (n = 46), sensitivity and NPV were slightly lower with FAPI. Among 59 T-regions surgically examined, malignancy was excluded in 6/59 (10%). FAPI showed (vs. FDG): sensitivity 85% (vs. 72%), specificity 67% (vs. 50%), PPV 96% (vs. 93%), NPV 33% (vs. 17%), accuracy 83% (vs. 69%). Among 217 N-stations surgically assessed (overall 746 ln removed), only 15/217 (7%) resulted malignant; FAPI showed (vs. FDG): sensitivity 53% (vs. 60%), PPV 53% (vs. 26%), NPV 97% (vs. 97%), and significantly higher specificity (97% vs. 88%, *p* = 0.001) and accuracy (94% vs. 86%, *p* = 0.018). Semiquantitative-PET parameters performed similarly, better for N (*p* < 0.001) than for T, slightly in favour (although not significantly) of FAPI over FDG. Conclusions: In a suspected/confirmed lung cancer surgical cohort, PET/CT performances for preoperative T&Nstaging were slightly in favour of FAPI than FDG (except for suboptimal N-sensitivity), significantly better only for N (region-based) specificity and accuracy using visual assessment. The trial’s conventional follow-up is still ongoing; future analyses are pending, including non-surgical findings and theoretical impact on patient management.

## 1. Introduction

Despite the widespread screening measures and imaging methods with their steadily increasing diagnostic accuracy, lung cancer remains the first tumour for mortality in both genders [1,2].

Although [18F]-Fluorodeoxyglucose (FDG) is a routinely used PET/CT tracer for cancer staging, it is affected by known performance limitations, and it is not suitable for theranostic (therapeutic and diagnostic) purposes. In staging lung cancer, FDG-PET/CT can be limited in detecting microscopic tumour deposits and non-FDG-avid histotypes such as mucinous neoplasms and lepidic predominant adenocarcinomas [3]. Currently, diagnostic chest CT remains the modality of choice for the evaluation of T in lung cancer, but in mediastinal staging (N), FDG-PET/CT resulted superior [4]. Nevertheless, especially in N staging, FDG-PET/CT can be, in a non-negligible number of patients, either false negative (FN) in small (<1 cm) or colliquative/necrotic lymph node (ln) metastases (LNM) or false positive (FP) in inflammatory/granulomatous/infectious processes [5].

Endobronchial ultrasound-guided transbronchial needle aspiration (EBUS-TBNA) is the “gold standard” for the evaluation of mediastinal and hilar ln, but its diagnostic accuracy is still insufficient, mainly due to intratumor heterogeneity, endoscopist skills’ variability, impossibility to assess specific ln stations (i.e., 5, 6, and 9) [6,7]. Furthermore, TBNA remains an invasive method associated with patient discomfort, sedation, and complications (i.e., bleeding, pneumothorax, infection).

Currently, anatomical lobectomy, along with systematic ln dissection (LND), is considered to be the main surgical approach. For example, for early-stage non-small cell lung cancer-(NSCLC) [8], nevertheless, for patients without LNM, LND turns out to be excessive, extending surgical duration and increasing perioperative complications.

The empowerment of an additional non-invasive modality becomes crucial for accurate staging.

Cancer-associated fibroblasts (CAFs), which support angiogenesis, invasive metastasis, and tumour growth, are one of the key components of the tumour microenvironment and a pivotal factor in cancer progression.

The fibroblast activation protein (FAP), a type II transmembrane glycoprotein on CAFs, is overexpressed in various malignancies, characterised by a strong desmoplastic reaction that can contribute up to 90% of the tumour mass [9,10,11]. Therefore, FAP inhibitors (FAPI), specifically binding to FAP, have been synthesised and modified to radiolabelled (i.e., with 18F or 68Ga) FAPI for PET imaging [12,13,14,15].

Some important tracer characteristics are a fast renal clearance, a high tumour-to-background ratio (TBR), a favourable biodistribution in the liver, adrenal glands, brain (i.e., frequent sites of lung cancer metastases), heart, bowel and a potentially shorter uptake time compared to FDG (high and comparable lesion detection at both 10 and 60 min [16]. Several FAPI molecules have been explored so far. In particular, PET/CT performed with FAPI-46 already demonstrated a favourable dosimetry profile [17].

FAP is upregulated in lung cancer as well, varying by subtype [18]. NSCLC shows FAP expression levels of up to 100% (in 344 NSCLC tissues examined [19], overall, FAP expression in tumour cells and its combination in tumour cells and CAFs were strongly associated with patient survival as useful predictive biomarkers for outcome), whereas small cell lung cancer and large cell neuroendocrine carcinoma are known to express FAP biomarker in up to 67%.

Taking into account the preliminary encouraging results of [68Ga]-Gallium-Fibroblast-Activation-Protein-Inhibitor (FAPI) PET/CT across various cancer types [20,21,22,23,24,25,26], it becomes crucial to analyse the performance of this promising imaging tracer when compared with the best imaging method that has been available so far (FDG-PET/CT) in specified clinical contexts, such as staging lung cancer.

Therefore, a prospective monocentric study (EudraCT number: 2021-006570-23; CE AVEC: 51/2022/Farm/AOUBo) was designed to investigate the performance of FAPI-PET/CT in 60–80 patients with suspected/newly diagnosed lung cancer in staging, already scheduled for a conventional staging flow-chart (including FDG-PET/CT), with no changes in patient’s management deriving from FAPI (referring surgeon and patients blinded to FAPI results): in patients undergoing surgery FAPI PET/CT findings were validated by pathology and 1-year conventional follow-up; in patients excluded from surgery, concordance with conventional staging imaging was assessed.

For the current analyses, only the surgical cohort was selected.

## 2. Materials and Methods

### 2.1. Study Design

A prospective monocentric explorative study was designed to enrol a consecutive series of approximately 60–80 patients, satisfying all the following criteria (specified below), to investigate the performance of 68Ga-FAPI-46 (FAPI) PET/CT in staging patients with suspected/confirmed lung cancer. This research project is funded by FIN-RER 2020 Programme of Emilia-Romagna Region (Grant code: FIN-RER_BU_2020_46).

#### 2.1.1. Inclusion Criteria

(a) Patients with suspected/newly diagnosed lung cancer, already scheduled for a standard staging diagnostic flow-chart (including FDG-PET/CT); (b) age ≥18; (c) both genders; (d) signed informed consent.

#### 2.1.2. Exclusion Criteria

(a) Pregnancy; (b) breastfeeding; (c) emergency situations or unfit to plead; (d) history of allergic reactions or hypersensitivity to the active substance, to any of the excipients, or to any of the components of the radiolabelled radiopharmaceutical; (e) contraindication to PET/CT examination for patients unable to perform PET due to weight, claustrophobia or the inability to remain still for the duration of the examination; (f) participation in a clinical trial in which an investigational drug was administered within 30 days or 5 half-lives before the study drug; (g) severely impaired renal function; (h) severely impaired liver function.

The study was conducted according to Good Clinical Practices (GCP) after local Ethical Committee (EC) and AIFA (Associazione Italiana del Farmaco) approval (EudraCT number: 2021-006570-23; CE AVEC: 51/2022/Farm/AOUBo).

Each patient was enrolled at Nuclear Medicine Unit (NMU) to undergo a FAPI-PET/CT in addition to the conventional staging flow-chart (including FDG-PET/CT performed at NMU, referred by the Division of Thoracic Surgery-DTS and/or Interventional Pulmonology Unit-IPU of IRCCS AOUBO), with no changes in patient management, nor delay in standard diagnostic and therapeutic work-flow deriving from FAPI results. The treatment plan was decided by the referring clinicians on the basis of a standard clinical and imaging staging flowchart [27]: the majority of patients were expected to be addressed to surgery; on the other hand, only a small percentage was expected to be excluded from surgery [28]. Patients referred to surgery underwent standard pathology evaluation and were routinely monitored (by DTS) for 1 year according to standard practice [29].

### 2.2. Aim of the Present Analyses

Only the surgical cohort was included and analysed in the present paper for the following purposes.

The primary aim was to investigate the preoperative diagnostic performance of the investigational FAPI-PET/CT for T&N on a patient and region-based evaluation in the setting of suspected/confirmed lung cancer patients addressed (by conventional staging work-up only) to surgical histopathological assessment.

The secondary aims were: (i) to investigate the pre-operative diagnostic performance of semi-quantitative parameters of FAPI uptake, (ii) to investigate the agreement (%) between FAPI/FDG and histopathology (by TNM-StagingAJCC8thEdition); (iii) to explore the association of FAPI uptake at PET/CT with immunohistochemical (IHC) FAP-expression on the histopathological samples.

### 2.3. Radiosynthesis of [68Ga]-FAPI-46

[68Ga]-FAPI-46 was synthesised by the local radiopharmacy (NMU), in accordance with the Italian regulations for hospital preparation and quality control of radiopharmaceuticals, in a fully-automated synthesis module (PharmaTracer, Eckert and Ziegler, Berlin, Germany) using the eluates of 2 GalliaPharm generators (1850 MBq, Eckert and Ziegler, Berlin, Germany). Briefly, radiolabelling was performed by heating to 95 °C a mixture of 40 nmol of FAPI-46 precursor (SOFIE, Dulles, VA, USA), ascorbic acid, 68GaCl3 and acetate buffer (pH = 4.5) for 15 min. Afterwards, the product was purified by a C18 SPE cartridge and eluted with EtOH in a vial containing Vitamin C (Bayer, Milan, Italy) in saline. The final product was diluted with saline and sterilised by passing through a 0.22-μm filter before dispensing. The radiochemical purity was over 95%, and the final product was sterile and pyrogen-free [30].

### 2.4. FAPI Procedure

Each patient was subjected to an intravenous injection of approximately 170 MBq of FAPI-46. Images were acquired on hybrid PET/CT Tomographs after 60 min of uptake. Non-diagnostic low/intermediate dose CT was performed both for attenuation correction and as an anatomical mapping. Scans were performed from the head vertex to the upper thighs.

### 2.5. PET/CT Imaging Review

Investigational FAPI-PET/CT was performed at NMU and interpreted by at least 2 independent nuclear medicine physicians (L.Z., E.F.) with high expertise in oncological PET/CT reading. In case of disagreement, consensus was reached by the opinion of further readers (G.C., C.N.).

PET/CT images were evaluated on a dedicated workstation (Philips-Vue-Picture archiving and communication system—PACS software v 24.7). Visual-positivity-criteria was defined, for T&N, as uptake (other than physiological/para-physiological distribution) visually significantly higher than the surrounding background activity and generally above mediastinal blood pool (MBP), and interpreted for the likelihood of presence/absence of disease taking into account both functional characteristics, morphological aspect and site. The size of T and of target N were reported.

Region-based analyses were classified based on the lung anatomical segments for T and on the Regional Lymph Node Classification System for N [31,32,33,34].

Areas of significant uptake outside the thorax were also encountered but excluded from the present analyses, being out of the topic (exclusively on the surgical findings).

A semi-quantitative analysis was performed to help the PET reading: a volume of interest (VOI) was considered on the T&N avid area to calculate standardise uptake values (SUVmax and SUVmean). Tumour-to-background-ratios (TBRs) were also calculated dividing the target T&N SUVmax by either SUVmax or SUVmean of different reference backgrounds, such as MBP (1 cm^3^-VOI within the thoracic aorta arch lumen), liver (L) (3 cm^3^-VOI in healthy hepatic parenchyma; when possible, in the right lobe), and pulmonary parenchyma (P) (1.5 cm^3^-VOI in the healthy lung).

Both patients and referring physicians were blinded to the report of FAPI-PET/CT, therefore, with no changes in patient management nor delay in standard diagnostic and therapeutic flow-chart deriving from investigational FAPI-PET/CT results.

In participants who were addressed (according to the standard of care) to surgery, PET/CT results (T&N) were validated by histopathological analysis (routinely processed) of the surgical specimens, used as a standard of truth to define scan results, per patient and per region, as true positive (TP), true negative (TN), FP or FN.

### 2.6. Histopathological and Immunohistochemical Analyses

The surgical plan was decided and performed by experienced surgeons (DTS, IRCCS, AOUBO) following standard recommendations [8], according to clinical and conventional imaging staging work-up and not altered by the results of the investigational scan. The histopathological examinations of the surgical specimens were all performed by Pathology IRCCS, AOU di Bologna, as part of standard practice and in accordance with European guidelines. At least one experienced, dedicated pathologist (F.G.) reviewed all cases.

Regarding immunohistochemical (IHC) analyses, additional immunostaining for FAPi expression was performed (A.D.) using the following procedure.

All samples were fixed in formalin and embedded in paraffin. From the paraffin blocks, 3-μm-thick sections were cut. The study was conducted with an automatic immunohistochemistry stainer, Benchmark Ultra (Ventana/Roche Group, 1910 Innovation Park Dr., Tucson, AZ, USA). The immunostaining for FAPI/MUM1 was performed with antigen retrieval using Cell Conditioning 1 for 48 min at 100 °C. The primary antibody FAPI (dilution 1/200, Polyclonal AbCam, Cambridge, UK) was incubated for 16 min at 36 °C, and the secondary antibody MUM1 (clone EP190, prediluted, Roche, Monza, Italy) was incubated for 24 min at 36 °C. The revelation system used was OptiView DAB (12 min linker and 12 min HRP multimer), the OptiView Amplification Kit (Ventana/Roche) for FAPI, and Ultraview Red for MUM1. Counterstaining was performed with Hematoxylin II (Ventana/Roche) for 4 min.

For the analysis of FAPi positivity in the obtained images, the open-source artificial intelligence (AI) system QuPath was utilised. Annotations were created to outline the perimeter of the tumour areas, ensuring accurate localisation for the percentage quantification of marker-positive cells. The use of the “positive cell detection” setting enabled the count to focus exclusively on cells not marked with MUM1.

**Figure 1:**
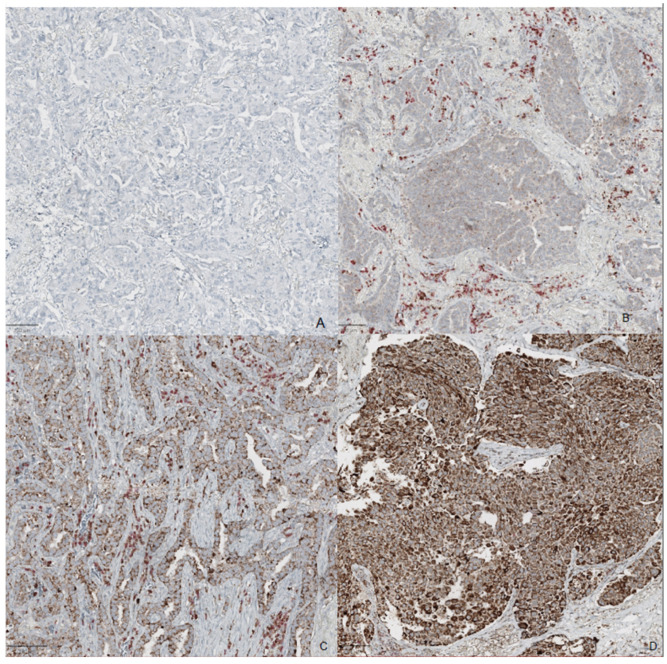
**FAPI immunohistochemistry.** Four-tiered system score: (**A**) score 0: negative stain, (**B**) score 1+: weak positivity in few neoplastic cells. There are plasma cells MUM 1 positive with red stain and double positivity with FAPI and MUM1 co-expression. (**C**) score 2+: with moderate positivity in 20–50% of cells. (**D**) score 3+ with strong positive stains in >50% of the cells.

Furthermore, a semiquantitative scoring system (Figure 1) was used to evaluate the amount of FAPI neoplastic-positive cells and fibroblastic-positive cells. The FAPI expression was defined through a four-tiered system score, according to the intensity of the stain, as follows: 0 = negative, 1+ = weak (>10% positive cells), 2+ = moderate (20–50% positive cells), and 3+ = strong (>50% positive cells). The presence of plasma cells with double positivity for FAPI/MUM1 was also assessed.

### 2.7. Data Comparison and Validation

The rationale for this approach was the comparison of the investigational FAPI imaging with standard clinical practice. All the conventional imaging scans were scheduled clinically and reimbursed as part of their standard clinical care and medical records.

FAPI results were compared with the standard of truth of histological findings in surgical specimens and were also evaluated in comparison to the tracer routinely available, FDG.

Visual Diagnostic performance for T&N was analysed per patient and region; diagnostic performances of semiquantitative PET parameters (receiver operating characteristics-ROC area under the curve-AUCs for SUVmax and TBRs) were evaluated region-based.

Agreement (%) between FAPI/FDG and histopathology was assessed by TNM-StagingAJCC8thEdition.

Associations between FAPI uptake and FAPI IHC were explored.

### 2.8. Statistical Analyses

Data management was done using a dedicated project platform, Redcap [35].

The analyses were carried out with the statistical software R (version 4.3.1) [36] using *p* < 0.05 as a threshold of statistical significance.

The characteristics of the patients enrolled in the study were reported in summary tables. Descriptive analyses were used to describe the collected data: continuous variables were reported by means, standard deviation (or median or interquartile distance), minimum and maximum values and percentiles, and discrete or nominal variables were described and summarised by frequencies absolute and relative percentage frequencies.

Accuracy, sensitivity and specificity for T&N were calculated, with relative 95% confidence intervals (95% CI), positive predictive value (PPV) and negative predictive value (NPV) of PET/CT with experimental FAPI and with standard FDG, both in a per patient and in a per station/region analysis. The same was calculated (region-based) for continuous semiquantitative PET variables (SUVmax, TBRs).

FAPI-PET/CT visual and semiquantitative performances for T&N (ROC AUC for SUVmax and TBRs) were analysed per patient and per region, compared to FDG-PET/CT, with surgical-histopathology as the standard of truth. Semiquantitative PET parameters’ AUCs were compared to each other and between the two tracers, FAPI/FDG, to assess statistical significance. The best cut-off values (Youden Index) to detect either malignant lung (T) or malignant hilar-mediastinal ln (N) were also searched.

The characteristics of the participants subjected to the two radiopharmaceuticals FDG-FAPI were compared using the chi-square test, Mann–Whitney test depending on the distribution of the examined variables.

Wilcoxon rank sum test was applied between semiquantitative PET parameters (SUVmax, TBRs) and benign/malignant groups.

The concordance index between the experimental FAPI and standard FDG was also evaluated using Cohen’s Kappa test (coefficient k).

The association between IHC FAPI expression (four-tiered system score previously mentioned or dichotomised into 0–1 vs. 2–3) either on T&N neoplastic cells, fibroblasts or plasma cells and 68Ga-FAPI-46 uptake (T&N-SUVmax and SUVmean) was explored with Wilcoxon rank sum tests. Chi-square test and/or Fisher were applied between IHC and visual FAPI PET/CT results or surgical histopathological results (neg/pos).

## 3. Results

63 FAPI scans were performed in 64 patients enrolled between 26 May 2022 and 30 November 2023 (1/64 screening failure: the patient signed informed consent to participate but finally dissented before Fapi administration). The tracer was well tolerated. No clinically significant adverse events were reported by patients.

Overall, 50/63 patients (mean age: 72 years, range 45–87; M:F = 33:17) underwent thoracic surgery (according to standard practice) and were included in the present analyses.

Per patient, a median of 173 MBq of 68Ga-FAPI-46 was injected intravenously (mean 173; sd 18; range 117–221; se 2.61).

The additional FAPI scan was performed approximately 2 weeks after the standard FDG scan, with a median of 13.5 days (mean 13.4; sd 10; range 0–34; se 1.42).

Patients underwent surgery mostly within 2 months from the experimental imaging, with a median of 40.5 days (mean 45.50; sd 30.38; range 7–154; se 4.30).

### 3.1. Agreement (%) with Histopathological-T&N-StagingAJCC 8th Edition

Surgery excluded malignant disease in 6/50 patients (4 T0N0; 2 T0Nx), whereas confirmed lung cancer in the remaining 44/50 patients (7 T1N0, 2 T1N1, 1 T1N2, 1 T1Nx; 14 T2N0, 4 T2N1, 1 T2N2, 1 T2Nx, 7 T3N0, 2 T3N1, 1 T3N2, 2 T4N0, 1 T4N2).

The summary (in terms of n° and %) of presentations of T (from T0 to T4) & N (from N0 to N3) by TNM-staging-AJCC8thEdition is presented separately in Table 1 for standard FDG and experimental FAPI PET/CT imaging and for the histopathological results from thoracic surgery (as gold standard).

The concordance of PET/CT with surgical histopathological results was assessed for T and resulted 66% for FAPI and 58% for FDG. The agreement for N was also in favour of FAPI, with 78% and 70%, respectively. When T&N was dichotomised into negative versus (vs.) positive for malignancy (instead of categorised according to StagingAJCC 8th ed.), agreement increased for T to 92% with FAPI and 80% with FDG respectively, for N to 78% and 72%, respectively (Table 2).

### 3.2. PET/CT Diagnostic Performance T&N, per Patient

Per patient, PET/CT diagnostic performance of visual criteria for T (n = 50) resulted favour of FAPI (although not significantly, in particular (FAPI vs. FDG), TP 42 vs. 37, FN 2 vs. 7, FP 2 vs. 3, TN 4 vs. 3; sensitivity 95% vs. 84%, specificity 67% vs. 50%, PPV 95% vs. 93%, NPV 67% vs. 30%, accuracy 92% vs. 80, respectively (Table 3).

Among the 50 patients of the surgical cohort, 46 were subjected to lymphadenectomy, resulting 34 pN0 and 12pN+ (26%; 8pN1 and 4pN2). PET/CT diagnostic performance of visual criteria for N (n = 46) resulted (FAPI vs. FDG): TP 6 vs. 8, FN 6 vs. 4, FP 4 vs. 9, TN 30 vs. 25; sensitivity 50% vs. 67%, specificity 88% vs. 74%, PPV 60% vs. 47%, NPV 83% vs. 86%, accuracy 78% vs. 72% (Table 3).

### 3.3. PET/CT Diagnostic Performance T&N, per Region

#### 3.3.1. PET/CT Visual Criteria

Among the 50 patients of the surgical cohort, 59 lung lesions (T) were examined (Table 4). 6/59 resulted benign: 1 actinomyces and 1 granulomatous abscess (concordant FDG&FAPI-FP), 4 inflammatory nodules (1 FDG-FP/FAPI-TN and 3 concordant-TN). 53/59 resulted malignant: 37 adenocarcinomas (7 concordant FDG&FAPI-FN, predominantly lepidic and in 1 case mucinous; 6 FDG-FN/FAPI-TP; 24 concordant FDG&FAPI-TP- the majority acinar), 13 squamous cell carcinoma-SCC (1 FDG-FN/FAPI-TP; 12 concordant FDG&FAPI-TP), 1 carcinoid (concordant FDG&FAPI-FN), 2 lung cancer with histology/histotype not specified (concordant FDG&FAPI-TP).

The 13 lung lesions (T), which resulted adenocarcinoma FN at PET/CT, presented mainly mixed subtypes, interestingly including lepidic and mucinous components. There were no statistically significant differences in the performance of the adenocarcinoma subtype. However, we observed that FDG had more FN compared to FAPI, which conversely presented more TP, especially for the lepidic subtype: 8 (FN) and 9 (TP) for FDG, 5 FN and 12 TP for FAPI; acinar subtype: 11 FN and 15 TP for FDG, 6 FN and 20 TP for FAPI; solid subtype: 4 FN and 10 TP for FDG, 14 TP for FAPI.

PET/CT diagnostic performance of visual criteria, region-based, for T (n = 59) resulted favour of FAPI (although not significantly), in particular (FAPI vs. FDG): sensitivity 85% vs. 72%, specificity 67% vs. 50%, PPV 96% vs. 93%, NPV 33% vs. 17%, accuracy 83% vs. 69% (Table 5).

Among the 46 patients who were addressed to nodal dissection according to standard practice, 746 lymph nodes were surgically resected overall, processed and examined, with a median of 12.5 for each patient (mean 16.2; range 2–60; 25°pctl 9.2; 75°pctl 21.7). Only 42/746 ln resulted secondary lesions (LNM) of lung cancer; in particular, for each patient, less than 1 ln resulted malignant (mean 0.9, median 0, range 0–18, 25°pctl 0; 75°pctl 0.75). Considering the regional lymph node classification system for N [31], overall, 217 N-stations were examined: only 15/217 (7%) resulted lung cancer metastases; 202/217 (93%) were not malignant/inflammatory.

PET/CT diagnostic performance of visual criteria, region-based, for N (n = 217) showed (FAPI vs. FDG): sensitivity 53% vs. 60%, specificity 97% vs. 88%, PPV 53% vs. 26%, NPV 97% vs. 97%, accuracy 94% vs. 86% (Table 5).

To note that statistical significance in favour of FAPI, using visual Criteria, was found only for N-region based (not per patient, nor for T) in terms of specificity, accuracy, number of TN and FP.

#### 3.3.2. Semiquantitative PET Parameters

FDG and FAPI SUVmax and mean values of the chosen reference backgrounds (MBP, L, P), measured to calculate the corresponding TBRs, were reported in detail in the supplementary Table S1: FDG and FAPI uptake of reference backgrounds within the surgical cohort (n = 50).

Semiquantitative PET parameters (SUVmax, TBRs) were significantly higher in the malignant group than in the benign group, with both tracers, for N (FAPI *p* < 0.0001; FDG-TBRs *p* < 0.0001 and FDG-SUVmax = 0.001), but not significantly different for T.

**Figure 2:**
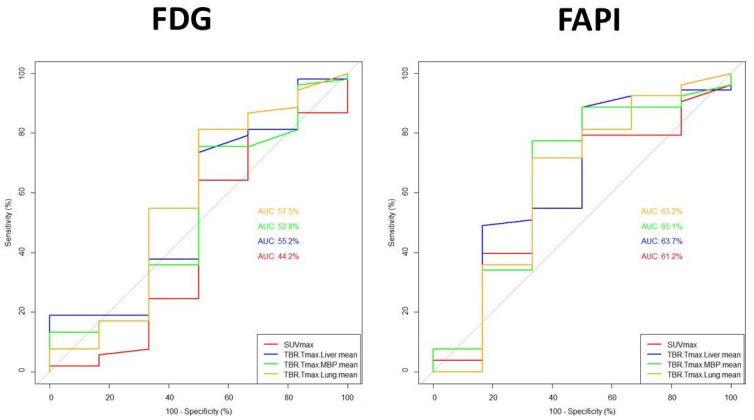
Comparison of Receiver operating characteristic (ROC) curves of PET/CT diagnostic performance using semiquantitative PET parameters (SUVmax, TBRs), region-based, for T (n = 59). The sub-figure on the left side shows the ROC curves for each FDG PET parameter in comparison. The sub-figure on the right side presents the ROC curves of each FAPI PET parameter in comparison. The area under the curves (AUC in %- see also Table 6; a colour legend in the bottom right side of each sub-figure) were not statistically significantly different between FDG parameters nor between FAPI parameters.

**Figure 3:**
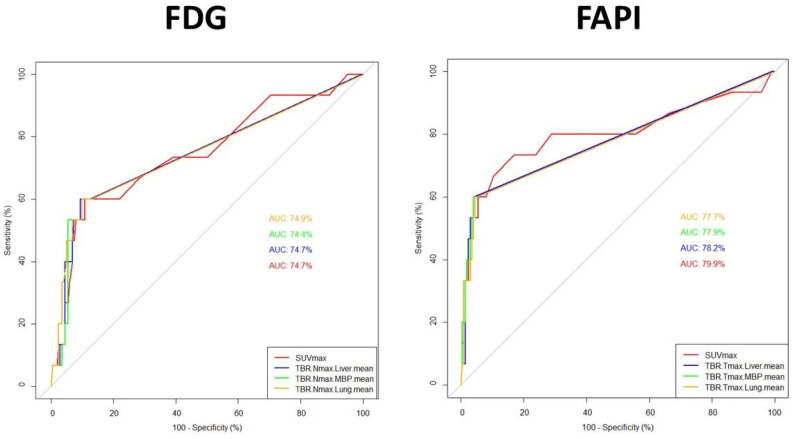
Comparison of Receiver Operating characteristic (ROC) curves of PET/CT diagnostic performance using Semiquantitative PET parameters (SUVmax, TBRs), region-based, for N (n = 217). The sub-figure on the left side shows the ROC curves for each FDG PET parameter in comparison. The sub-figure on the right side presents the ROC curves of each FAPI PET parameter in comparison. The area under the curves (AUC in %-see also Table 7; a colour legend on the bottom right side of each sub-figure) were not statistically significantly different between FDG parameters nor between FAPI parameters.

SUVmax, TBRs parameters performed similarly, better for N (AUCs *p* < 0.001) than for T, slightly in favour (although not significantly) of FAPI over FDG (Table 6 and Table 7; Figure 2 and Figure 3).

For T (n = 59) (FAPI vs. FDG, Table 6, Figure 2): SUVmax-AUC = 61.2% (cut-off = 2.1, accuracy = 72%) vs. 44.2% (cut-off = 12, accuracy = 76%), TBR-L-AUC = 63.7% (cut-off = 0.9 accuracy = 84%) vs. 55.2% (cut-off = 0.9 accuracy = 71%), TBR-MBP-AUC = 65.1% (cut-off = 2.3, accuracy = 76%) vs. 52.8% (cut-off = 1, accuracy = 73%), TBR-P-AUC = 63.2 (cut-off = 6.5, accuracy = 71%) vs. 57.5% (cut-off = 4, accuracy = 78%).

For N (n = 217) (FAPI vs. FDG, Table 7, Figure 3): SUVmax-AUC = 79.9% (cut-off = 1.5, accuracy = 82%) vs. 74.7% (cut-off = 3, accuracy = 87%), TBR-L-AUC = 78.2% (cut-off = 1.1; accuracy = 94%) vs. 74.7% (cut-off = 1.4, accuracy = 88%), TBR-MBP-AUC = 77.9% (cut-off = 1.7, accuracy = 94%) vs. 74.4% (cut-off = 1.7, accuracy = 88%), TBR-P-AUC = 77.7% (cut-off = 3.2, accuracy = 93%) vs. 74.9% (cut-off = 5.4, accuracy = 88%).

### 3.4. FAPI Uptake and FAP-IHC Expression

Statistically significant associations were found: between IHC expression of FAPi on plasma cells and the results of visual FAPI PET/CT (*p* = 0.0232); between IHC expression of FAPi on fibroblasts and the surgical histopathological result (*p* = 0.01428).

## 4. Discussion

### 4.1. Study Results

Lung cancer, for its incidence, morbidity, and mortality rate, still represents one of the most impacting oncologic diseases in the Health System. A diagnostic tracer able to identify lung cancer localisations with higher accuracy at the time of initial presentation could provide a more informed and directed treatment plan that would improve patient outcomes, reduce costs in terms of diagnostic procedures to reach the final diagnosis and reduce complications from invasive procedures. FAPI may represent a useful tool to overcome FDG drawbacks, being reproducible (in particular, the form labelled with 68Gallium could be easily available also in small nuclear medicine centres due to its easy and cyclotron-independent production), with safe radiation dosimetry and to the best of our knowledge, no major adverse events reported to date.

In 2020, at the time of the study hypothesis and design, despite promising results on FAPI PET/CT arising within the scientific community, the major limitations of the main studies were the small number of patients and the heterogeneity of cancer types included due to the preliminary explorative nature of the projects and to the innovation of this new tracer potentially able to detect several malignancies. Moreover, patients in previous studies were recruited at different stages of the natural history of the disease. Therefore, our research team decided to select only one specific cancer setting: staging suspected/confirmed lung cancer. Additionally, the present analyses were focused only on patients addressed to surgery in order to ensure a region-based T&N postoperative histopathological confirmation on virtually all patients.

Though we did not expect FAPI to replace FDG, we expected a complementary role with improved sensitivity in lung cancer histotypes with typically low metabolic activity [37] (such as lepidic lung cancer or, less frequently, other acinar- or papillary-dominant adenocarcinoma) and in small hilar-mediastinal ln metastases-LNM (typically FDG FN), and higher specificity in inflammatory ln and in general in cases of concomitant respiratory tract affections/pneumonia/granulomatosis (well-known FDG pitfalls) [38].

In our selected surgical cohort, for preoperative T&N staging suspected/confirmed lung cancer, FAPI finally revealed slightly better specificity and overall diagnostic performance (except for N sensitivity, slightly inferior/equal to FDG). In particular, a statistically significant difference between the two tracers, in favour of FAPI, was found in FP, TN, specificity, and accuracy for N (region-based) using visual criteria.

Due to the low N sensitivity (per patient = 50%, per region = 53%), comparable to a coin flip, in this preoperative setting, FAPI PET/CT does not reach clinically acceptable LNM detection or capability to rule out invasive but more accurate diagnostic procedures (such as EBUS-TBNA or LND) based on conventional staging and risk factors.

It is also well known that the wide variability and overlap between the size of non-metastatic and metastatic ln in conventional staging: LNM are often characterised by small, millimetric cancer deposits, expected to have low uptake due to the partial volume averaging and limited PET spatial resolution. It is unlikely, in the short term at least, that any imaging test will replace EBUS-TBNA and LND. More strengths should be addressed in relation to risk stratification and prognostication.

Region-based FNs were less with FAPI than with FDG for T (8 vs. 15) and similar for N (7 vs. 6).

As also reported for 68GaFAPI-04 [39], we can speculate that T&N 68GaFAPI-46 FN were mainly due to small dimension, low solid component (amount of necrosis/colliquation/ground-glass/lepidic component) and micro-metastases.

For example, in the study by Zohu et al. [40], three more FN LNM were missed when diagnosed by [68 Ga]Ga-DOTAFAPI-04 compared with 2-[18F]FDG, which might be attributed to the low FAP expression in small metastases [29]. Hence, the application of FAPI in the differential diagnosis of LNM still requires further verification in a larger number of patients.

**Figure 4:**
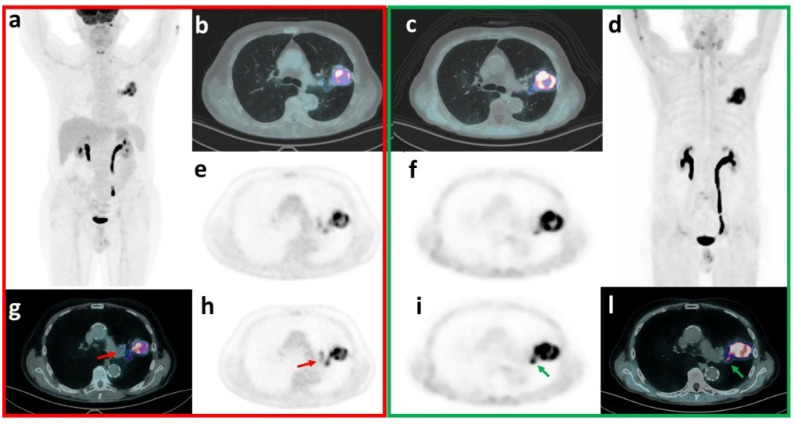
**T-False positive (FDG-FAPI concordant): granulomatous abscess.** 73 y.o. male patient underwent a thoracic diagnostic CT to investigate the presence of a lung lesion after the onset of haemoptysis, which detected a voluminous, solid and inhomogeneous lung lesion in the left superior lobe. FDG PET/CT showed intense and inhomogeneous uptake (SUVmax = 14.4) in the known lung lesion (**a**), FDG-MIP-maximum intensity projection; (**b**), transaxial PET fused with the attenuation correction-CT [ACCT]; (**e**), FDG transaxial PET). A moderate uptake was detected in subaortic (#5, SUVmax = 3), left hilar (#11, SUVmax = 5.1), and left interlobar peribronchial (#11, SUVmax = 5.1; (**g**), red arrow on transaxial PET fused with the ACCT; (**h**), red arrow on FDG transaxial PET) lymph nodes (T2N2M0). FAPI PET/CT detected intense and inhomogeneous uptake (SUVmax = 20.9) in the lung lesion (**d**), FAPI-MIP-maximum intensity projection; (**c**), transaxial PET fused with ACCT; (**f**) FAPI transaxial PET) and focal uptake only in left interlobar peribronchial lymph nodal station (#11, SUVmax = 10.1, (**l**) green arrow on transaxial PET fused with ACCT; (**i**) green arrow on FAPI transaxial PET) (T2N1M0). Endobronchial ultrasound-guided biopsies resulted inconclusive in the lung and excluded malignancy in #4L (TXN0M0). Subsequent left superior lobectomy demonstrated necrotic inflammation with hilar abscess and contiguous areas of pneumonia. Inflamed lymph nodes were detected in #5, #7, #9, #10, #11. Immunochemistry was also performed with FAPI staining: immunoreactivity in fibroblasts and plasma cells was detected.

In our series, regarding T, concordant FP findings with both tracers were 1 actinomyces abscess [41] and 1 granulomatous abscess (Figure 4). An inflammatory nodule resulted FP with FDG but TN with FAPI.

Regarding N, only 7 FP nodal stations were reported; on the contrary, 25 had FDG (*p* = 0.0018). Therefore, we can conclude that FAPI was able to reduce the number of FP in N-stations.

Despite yielding optimal specificity (FAPI vs. FDG 97% vs. 88%, *p=* 0.001) and accuracy (94% vs. 86%, *p* = 0.018) to identify malignant N stations, PPV was in favour of FAPI but still inadequate with both tracers (53% vs. 26%). In our opinion, this might be mainly a reflection of disease prevalence (only 12/46–26% of patients resulted pN+; only 15/217—7% N-stations resulted metastatic) and of reading criteria (visually significant accumulation of FAPI in fibrotic/granulomatous not malignant conditions does not completely overcome the critical issue of pitfalls/FP; algorithms combining further functional and morphological characteristics would help). In the literature, it was already reported that FAPI mimicked malignancies also, i.e., in pneumonia [42], Klebsiella pneumoniae invasion syndrome [43], epithelioid granuloma [44], multisystemic tuberculosis [45,46,47], sarcoidosis [48], granulomatosis by Francisella tularensis [49], pulmonary cryptococcosis [50].

**Figure 5:**
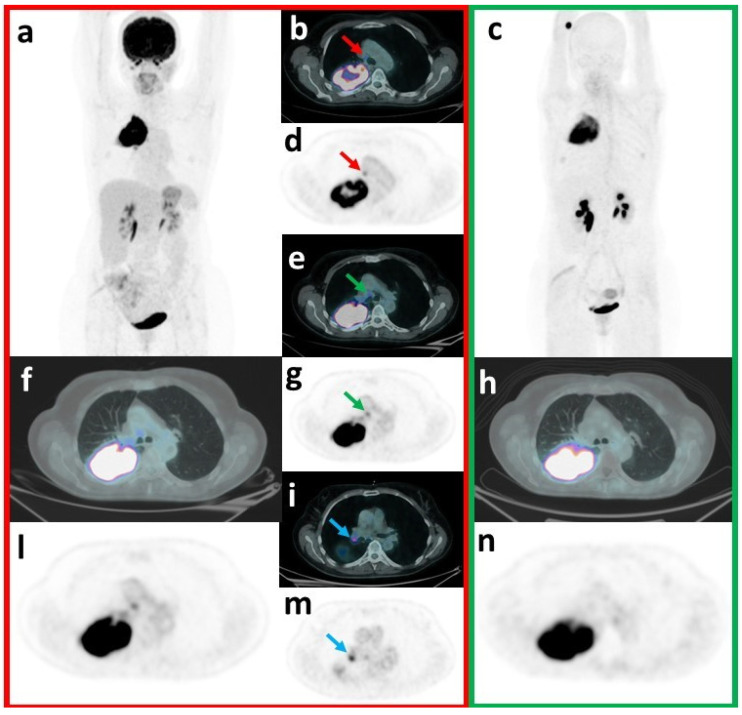
**N-FAPI True negative—FDG False positive.** A 60 y.o. woman underwent a diagnostic CT to investigate the onset of persistent cough, asthenia, weight loss, fever and haemoptysis. Diagnostic CT revealed the presence of a voluminous lung lesion in the right superior lobe, requiring PET/CT staging. FDG PET/CT detected intense and inhomogeneous uptake (SUVmax = 28.8) in the known lung lesion (**a**) FDG-MIP-maximum intensity projection; (**f**) transaxial PET fused with the attenuation correction-CT [ACCT]; (**l**) FDG transaxial PET). Significant uptake was also observed respectively in the right lower paratracheal (#4; SUVmax = 4.3; (**b**) red arrow on transaxial PET fused with the ACCT; (**d**) red arrow on FDG transaxial PET), subcarinal (#7, SUVmax = 4.9; (**e**) green arrow on transaxial PET fused with the ACCT; (**g**) green arrow on FDG transaxial PET) and right hilar ln stations (#10, SUVmax = 8.9; (**i**) blue arrow on transaxial PET fused with the ACCT; (**m**) blue arrow on FDG transaxial PET), suggestive for LNM (T3N2M0). FAPI PET/CT also confirmed the intense and inhomogeneous uptake in the lung lesion (SUVmax = 22.5; (**c**) FDG-MIP-maximum intensity projection; (**h**) transaxial PET fused with the ACCT; (**n**) FDG transaxial PET), but did not detect any significant lymph nodal uptake (T3N0M0). Endobronchial ultrasound-guided biopsies resulted positive in the lung for squamous cell carcinoma but negative for metastasis in the right lower paratracheal, subcarinal and right hilar lymph nodes. Surgery confirmed biopsies results: a poorly differentiated squamous cell carcinoma, with pleural, bronchial and vascular invasion, was diagnosed without lymph nodal involvement (pT3N0Mx). Subsequently, the patient started chemotherapy.

On the other hand, our data confirm the potential advantage of FAPI compared to FDG PET/CT in unmasking TN ln findings (N-per region, FAPI vs. FDG: 195 vs. 177, *p* = 0.019) (Figure 5). For example, FDG positive-FAPI negative anthracosis, due to macrophages rather than fibroblast activation, was already described [39,51].

Visual criteria represent a subjective process not directly adaptable and reproducible in clinical practice due to inter-reader variability, but we can speculate that each radiotracer is somehow dependent on reader experience. The novelty of our study is that, at the time of our project design and start, the attention was also focused on the role of several semi-quantitative functional parameters of the relatively new FAPI, whose interpretative criteria were not defined yet.

The liver represents an important background when it comes to FDG in the setting of aggressive lymphomas and multiple myelomas (i.e., to define Deauville score criteria) [52,53]. In our setting, the liver showed variable mild to moderate, heterogeneous FAPI uptake (L-SUVmax and SUVmean lower with FAPI than with FDG, but a wide range was shown—Table S1), thus representing a more complex reference background.

On the contrary, in favour of MBP background, we already know that it represents a reference structure for FDG in lung cancer imaging [54], and it is commonly used as a visual positivity criterion in the majority of published FAPI studies [37,55,56,57].

Performances of the investigational FAPI did not demonstrate a definite superiority of any particular PET parameter (SUVmax, TBRs): for T, AUCs ranging from approximately 61% of SUVmax to 65% of TBR-MBP (vs. FDG ranging from 44.4 of SUVmax to 57% of TBR-P); for N AUCs ranging approximately 78–80% (vs. FDG approximately 74%). To note that ROC-AUCs of each parameter resulted significant for N (*p* < 0.001) but not for T. Thus, the search for the best cutoff values to predict T malignancy should be considered only exploratory and not informative to support a definitive threshold, but potentially more reliable for N. Considering the calculated cut-offs to better discriminate malignancy: regarding T, to reach acceptable accuracy (>70%), the threshold for positivity criteria should approximately double MBP and be similar to L (significantly higher than P, SUVmax > 2); regarding N, to reach acceptable good accuracy (>80%), the cut-off should be triple than P, significantly higher than MBP (TBR 1.7), and equal to L (SUVmax > 1.5).

### 4.2. Limitations

Due to radiosynthesis requirements and the high burden of the diagnostic centre, to enable the higher number of patients scanned for each experimental session, simultaneous acquisitions of FAPI-PET were performed on the 4 available state-of-the-art PET/CT Tomographs [30]. FAPI PET/CT acquisition was scheduled on the same Tomograph used for the corresponding previous FDG for a head-to-head scan comparison to limit intrinsic different technical characteristics (when not possible for practical reasons, standard OSEM PET series were used for imaging review for both FDG and FAPI, to avoid differences in PET reconstructions). Note also that the short time interval between the two PET scans, the median inferior to 2 months of the time interval between imaging and surgery and the absence of neo-adjuvant therapy, potentially prevented any significant modification/evolution of the clinical-pathological disease status and helped a better results comparison between the different procedures.

It is known that ln templates adjacent to each other may be conflated between imaging and surgery. In addition, although standardised, variability in surgical attitudes may influence results. Moreover, the standard clinical need for patient management (i.e., the decision of LND extent) is the presence and anatomical N region location rather than the absolute number of affected ln. To reduce confounding factors, we excluded correlating PET/CT and histopathological results on a single ln level, and we chose to analyse nodal involvement on a patient and N region/station bases only. Each station was considered positive in case of 1 or ≥ 1 positive ln, which consists of a bias of our results since a per-node analysis was not performed. Only one representative ln from each field was correlated to histopathology, limiting the assessment in case of concomitant oncological and inflammatory findings in the same field. In the presence of more than 1 avid ln in the same region, the target ln with the highest uptake was selected.

The 8th edition of the AJCC TNM staging system was applied to imaging and histopathological findings, which was the official one during the trial enrollment. Recently, the 9th edition was proposed (including a subdivision of N2 and reordering of TNM staging groups), but it has not yet been effective [58]. The implementation of the new system in future analyses might also reveal changes in staging classification in our small population.

The study was not designed to directly compare PET/CT with conventional HRCT/CECT or EBUS-TBNA (data non included in the present analyses) but focused on the role of [68Ga]FAPI-46 PET/CT as a method to address the limitations of [18F]FDG PET/CT.

The interesting possibility of exploring non-surgical findings (i.e., N3/M+ patients) was also abandoned due to the lack of surgical pathological validation. Our selected population and, therefore, our results do not convey the global FAPI PET/CT performance in lung cancer.

Analyses are still ongoing. According to the project design, surgical patients will be routinely monitored by referring the thoracic group for 1-year after FAPI; this follow-up is still ongoing and will be used as a reference standard in order to validate PET results in sites not addressed to surgery. In patients excluded from surgery, the positivity rate will be calculated for both FAPI and conventional staging imaging (i.e., FDG PET/CT) in T/N/M staging, and concordance will be assessed. At the end of the standard management of enrolled patients, the conventional and already performed treatment plan (derived from the conventional staging flow-chart) and the hypothetical one (derived from the investigational tracer) will be compared to assess a potential clinical impact.

FAPI PET/CT represents a non-invasive tool to profile FAP expression, which becomes essential for theranostic purposes. However, the primary aim of our study was purely diagnostic (PET/CT performance for T&N); therefore, routine histopathological analyses of the surgical specimens were sufficient to validate PET results. FAPi immunostaining was additional, mainly descriptive data were recorded, and preliminary tests were applied to search for associations between IHC FAP expression in vivo and FAPI uptake on PET/CT. More specific analyses/discussions should be further developed within more dedicated/focused IHC studies.

We reached the lower limit (63 patients) of the established recruitment range (60–80 patients): indeed, a not negligible number of patients might not give consent to undergo an experimental procedure, in addition to the normal diagnostic flow-chart but with the blinded result not influencing his management and therefore with no direct benefit/advantage on his clinical history. This was a limiting step for enrolment and consecutivity.

However, the sample size of our surgical cohort is adequate, even larger than the main published studies to date, and reliable, given that the availability of surgical histopathology (for a T&N region-based performance analysis) was actually the inclusion criteria of the present paper.

On the other hand, the small proportion of patients with LNM within our surgical cohort may lead to statistical uncertainty.

The predominance of participants with adenocarcinoma may also limit the generalizability of the findings and their interpretation across the different pathologic lung cancer subtypes.

### 4.3. FAPI and Lung Cancer: Current Status

#### 4.3.1. FAPI and Lung Cancer

The first clinical studies on FAPI were explorative across different cancers.

Kratochwil et al. investigated 80 patients with 28 different tumour entities, including lung cancer, showing moderate to high uptake [21]. A study by Chen et al. compared FAPI and standard FDG-PET/CT for the diagnosis of primary and metastatic lesions in patients with different malignancies, reporting a higher FAPI TNM detection rate, a significantly higher uptake, a better visualisation of very small tumours (diameter < 1.0 cm) and a higher N sensitivity [22].

In 2023, there were already seventy-eight potentially eligible citations specifically on the selected topic of FAPI in lung cancer, but finally, only five articles were included in the meta-analysis by Yang et al. [59]: the overall sensitivity for T was 0.98 (95% CI: 0.88–1.00), vs. FDG 0.99 (95% CI: 0.74–1.00). For NM, FAPI PET/CT had a sensitivity of 0.99 (95% CI: 0.90–1.00) vs. FDG 0.77 (95% CI: 0.66–0.85).

To date, several different FAPI molecules have been investigated in lung cancer, labelled with 68Ga or 18F.

Sun et al. [60] investigated the value of FAPI labelled with 18F in diagnosing mediastinal and hilar lymph nodes (45/137 LNM) in lung cancer patients (n = 27), presenting much higher N sensitivity, specificity, accuracy, PPV and NPV than FDG (84% vs. 71%; 92% vs. 67%; 90% vs. 69%, 84% vs. 52%, and 92% vs. 83%, respectively), reaching a specificity of 96% (vs. 72%) in small ln. However, the final diagnosis was not confirmed by surgical histopathology but with EBUS or follow-up contrast-enhanced CT performed 6 months after treatment. Interestingly, two optimal values of FAPI-SUVmax were found: for specificity and PPV 5.3, whereas for sensitivity and NPV 2.5. These thresholds are not directly comparable with ours (Table 6 and Table 7), which were searched only for the best accuracy.

In a prospective trial enrolling 68 lung cancer participants with 548 lesions, Wei et al. [57] found that TBR-MBP was higher for N (7.5 ± 6.6 vs. 5.9 ± 8.6; *p* < 0.001) and bone metastases with 18F-FAPI-04 than FDG but lower for T (25.3 ± 14.0 [SD] vs. 32.1 ± 21.1; *p* < 0.001). For lung cancer diagnosis, FAPI had a higher sensitivity (99% vs. 87%; *p* < 0.001), specificity (93% vs. 79%; *p* = 0.004), accuracy (97% vs. 85%; *p* < 0.001), and NPV (97% vs. 70%; *p* < 0.001) but comparable PPV. The majority of patients (61/68) received a biopsy, but in the case of tumour lesions without biopsy, routine clinical and imaging follow-up were used. To note that when taking into account only surgical patients (7/68 patients with pathology, depicting 120 lesions) to reproduce our same condition, the performance values did not show any significant difference. Additionally, as in our series, FAPI uptake resulted significantly increased in a granuloma, which usually forms by infiltration of macrophages and their descendants, probably explaining FAPI false positivity.

Regarding IHC, Wei et al. demonstrated a moderate correlation between 18F-FAPI–derived SUVmax and FAP expression in 6 surgical and 26 biopsy lung cancer specimens [61].

As part of a retrospective analysis by the group of Quiao et al. [62], a total of 70 inflammatory and 37 malignant lung lesions were evaluated with [18F]AlF-NOTA-FAPI-04 PET/CT, and 33 inflammatory and 26 malignant lung lesions also with FDG. Benign lesions showed statistically significantly lower [18F]AlF-NOTA-FAPI-04 and FDG uptake compared to malignant (all *p* < 0.001). Interestingly [18F]AlF-NOTA-FAPI-04 uptake (SUVmax, SUVmean, and TBR-L) significantly varied among different types of inflammatory lesions, with the lowest uptake in pneumonia and the highest in infected bronchiectasis followed by post-obstructive pneumonia.

In a recent analysis by the group of Heidelberg (Germany) [37], all cases of lung cancer showed markedly elevated 68Ga-FAPI-46 uptake, increased TBRs, and increased 68Ga-FAPI46/FDG ratios for all parameters compared with benign pulmonary lesions. Interestingly nineteen patients underwent static and dynamic 68Ga-FAPI-46 PET in addition to FDG in order to characterise lepidic lung cancer as a known FDG–negative target, and FAP IHC of 24 tissue sections of lepidic lung cancer surprisingly revealed strong FAP positivity in all specimens.

In 10 patients with lung cancer, Giesel and collaborators [63] acquired PET scans at 10 min, 1 h, and 3 h after administration of 18F or 68Ga FAPI-74, resulting in high contrast and low radiation burden imaging. Centralised large-scale production of 18F-FAPI-74 or decentralised cold-kit labelling of 68GaFAPI-74 would allow flexible routine use.

#### 4.3.2. FAPI and NSCLC

A recent prospective Chinese study [64] by Wu et al. (28 NSCLC participants) showed a significantly higher TBR of 68Ga-FAPI over FDG and confirmed excellent N staging accuracy (80% [8/10]) (for M, out of the current topic, 92.9%, 26/28); to note that validation histopathology was lacking in many cases and that the minimum duration of follow-up for standard reference was only 3 months.

A prospective pilot study by Kang et al. [56], using the same type and amount of radiopharmaceutical [68Ga]FAPI-46 but with different positivity visual criteria (more than twice the intensity of MBP), was addressed to especially identify NSCLC N2 stage, a pivotal aspect in making management plan decisions for surgical candidates. In per-station analysis (23 patients, with 75 nodal stations, scheduled for surgery within 2 days), [68Ga]FAPI-46 PET/CT discriminated metastasis more effectively compared to FDG (AUC 0.96 (0.88–0.99) vs. 0.68 (0.56–0.78), *p* < 0.001).

A retrospective study by Li et al. (NCT05034146) [39] included 91 participants with suspected or biopsy-confirmed NSCLC who underwent 68Ga-FAPI-04 PET/CT for initial staging. Thirty-one patients underwent surgical LND, and 7 received EBUS-TBNA. For T, sensitivity resulted 96.7% and PPV 100%; for N station-based (n = 141, comprising 357 ln), sensitivity, specificity, and accuracy were 72%, 93%, and 89%, respectively. 68Ga-FAPI-04 uptake showed a close association with FAP expression, especially in terms of the volume parameters; the major cause of FN was nonsolid nodules (DR = 75%). However, strong FAP expression and high 68Ga-FAPI-04 uptake were also reported in benign conditions such as organising pneumonia, tuberculosis and cryptococcosis.

Another study, by Li et al. (ChiCTR2100044944) [55], on [18F])-labelled FAPI in NSCLC focused only on N performance in patients with stage I-IIIA, with surgical and pathological validation within 40 days. As in our [68Ga] FAPI setting, the N specificity of [ 18F]FAPI was significantly higher than FDG (*p* < 0.001). The optimal FAPI SUVmax cut-off for NPV/PPV (approximately 90%) was 6.2. N staging accuracies were 35.8% and 66.0% for FDG and FAPI, respectively, reaching 83% when integrating the two modalities. To note that, unlike our studies, negative lymph nodes were never sampled, which could have introduced bias in their performance values.

[68 Ga]Ga-DOTA-FAPI-04, investigated by Zhou et al. (ChiCTR2000038080) [40], showed better diagnostic performance than FDG in 79 lung nodules of 72 patients, especially for nonsolid and small-dimension adenocarcinoma, and in 98 ln of 37 participants (94% vs. 30%, *p* < 0.001; optimum cur-off 5.5). However, pathological results not only from surgical resection but also from biopsy within 2 months of PET/ CT were used as the gold standard, and several PET-positive lymph nodes were validated by follow-up examinations rather than pathology.

### 4.4. FAPI and Lung Cancer: Future Perspectives

Considering the significantly better FAPI specificity for N, integrating FDG and FAPI within a dual imaging approach would be a possible option, especially in the setting of malignant pulmonary diseases with confounding inflammatory processes [65]. However, to better discriminate the remaining pitfalls, acquiring delayed images or further exploring semiquantitative differential analysis should be considered [66]. New efforts should also be directed towards investigating the delineation of the radiotherapy field of view.

Diagnostic performance remains suboptimal in terms of N sensitivity. Therefore, there is still the need for a refined strategy to select patients who truly require invasive staging procedures such as EBUS-TBNA and LND.

The issue of change in patient management could be even more relevant for M staging when we consider that 10 to 25% of patients with primary lung cancer may show unknown metastases at staging time [28,67]. Despite not being evaluated in this paper, since there was no objective of our work, the assessment of more advanced patients affected by extra-thoracic nodal, skeletal, and visceral metastases would be of paramount importance for a more tailored treatment (i.e., to shift patients from thoracic surgery to multimodal and systemic therapies).

The optimal timing of FAPI PET to best differentiate between malignant/benign hopefully will be soon solved by the implementation of FAPI dynamic studies on total-body PET/CT [68].

Refinement of interpretative criteria, as well as reader training implementation, is also warranted to improve performance when investigating a new tracer; artificial intelligence (AI)–based methods could provide more objective image analysis [69,70]; radiomics and deep learning classifiers in discriminating normal/abnormal PET findings would be further explored [71,72].

Given the heterogeneity in the available data analyses due to different cancers and different lung cancers, future research should also focus on examining lung tumour subtypes individually.

Gallium-68 ([68Ga]) FAPI has been the radiotracer mainly investigated in most previous studies. However, the low elution dose and short half-life of [68Ga] are practical limitations. In contrast, [18F] is the most commonly used radionuclide in clinical practice due to its more abundant production, longer half-life, and higher spatial resolution. Therefore, we expect further development in [18F]-labelling.

FAPI promising diagnostic performance paved the way also to tailored radioligand therapy (RLT) [73], delivering the therapeutic isotope to the sites expressing FAP, thus enhancing new frontiers for lung cancer therapy.

Further large-scale multicenter prospective clinical trials are warranted.

## 5. Conclusions

In a suspected/confirmed lung cancer surgical cohort, PET/CT performances for preoperative T&N staging were slightly in favour of the experimental FAPI than standard FDG (except for N-sensitivity, which remains suboptimal), significantly better only in terms of specificity and accuracy for N (region-based) using visual assessment. SUVmax, TBRs were significantly higher in the malignant group than in the benign group, with both tracers, for N, but not significantly different for T. Semiquantitative PET parameters performed similarly, better for N (AUCs *p* < 0.001) than for T, slightly in favour (although not significantly) of FAPI over FDG.

The trial’s conventional follow-up is still ongoing; future analyses are pending, including the assessment of the non-surgical cohort/findings and the theoretical impact on patient management.

## Figures and Tables

**Table 1:** Summary of T&N presentations, according to StagingAJCC8thEdition (FDG, FAPI, Surgical histopathology).

T (n = 50)	T-FDG		T-FAPI		T-SURGERY		N (n = 46)	N-FDG		N-FAPI		N-SURGERY	
	n°	Mean	n°	Mean	n°	Mean		n°	Mean	n°	Mean	n°	Mean
**0**	10	20%	6	12%	6	12%	**0**	29	63%	36	78%	34	74%
**1**	11	22%	13	26%	11	22%	**1**	5	11%	6	13%	8	17%
**2**	19	38%	20	40%	20	40%	**2**	11	24%	3	7%	4	9%
**3**	7	14%	8	16%	10	20%	**3**	1	2%	1	2%	0	0%
**4**	3	6%	3	6%	3	6%							

**Table 2:** Agreement (%) with histopathological-T&N-StagingAJCC8thEdition (FDG vs. FAPI).

(According to TNM_AJCC 8th Ed.)	CONCORDANCE WITH T-Histopathology		CONCORDANCE WITH N-Histopathology	
	FDG	FAPI	FDG	FAPI
**% agreement**	58	66	69.6	78.3
**Cohen’s Kappa value**	0.435	0.536	0.384	0.447
**Cohen’s Kappa *p*-value**	<0.001	<0.001	<0.001	<0.001
(dichotomized: neg vs. pos)	**CONCORDANCE WITH T-histopathology**		**CONCORDANCE WITH N-histopathology**	
	**FDG**	**FAPI**	**FDG**	**FAPI**
**% agreement**	80	92	71.7	78.4
**Cohen’s Kappa value**	0.265	0.621	0.354	0.404
**Cohen’s Kappa *p*-value**	0.05	<0.001	0.013	0.0058

**Table 3:** PET/CT diagnostic performance of visual criteria (FDG versus FAPI), patient-based, for T (n = 50) and for N (n = 46).

Visual Criteria (Patient-Based)	T (n = 50)			N (n = 46)		
	FDG	FAPI	*p*-Value	FDG	FAPI	*p*-Value
**TP**	37	42	n.s.	8	6	n.s.
**FN**	7	2		4	6	
**FP**	3	2		9	4	
**TN**	3	4		25	30	
**Sensitivity**	84%	95%		67%	50%	
**Specificity**	50%	67%		74%	88%	
**PPV**	93%	95%		47%	60%	
**NPV**	30%	67%		86%	83%	
**LR+**	1.68	2.86		2.52	4.25	
**LR−**	0.32	0.07		0.45	0.57	
**Accuracy**	80%	92%		72%	78%	

Legend: TP = True positive; FN = False negative; FP = False positive; TN = True negative; PPV = positive predictive value; NPV = negative predictive value; LR = likelihood ratio; n.s. = not significant.

**Table 4:** Visual criteria diagnostic performance by histological classification.

Visual Criteria Performance by Histological Classification (n = 59)	T (n°)			FDG				FAPI			
				FN	FP	TN	TP	FN	FP	TN	TP
**Adenocarcinoma**	37	53	**Malignant**	13			24	7			30
**Squamous cell carcinoma**	13			1			12				13
**Carcinoid**	1			1				1			
**Lung Cancer (histology not specified)**	2						2				2
**Inflammatory nodule**	4	6	**Benign**		1	3				4	
**Granulomatous abscess**	1				1				1		
**Actinomyces abscess**	1				1				1		
	59										

Legend: TP = True positive; FN = False negative; FP = False positive; TN = True negative.

**Table 5:** PET/CT diagnostic performance of visual criteria (FDG versus FAPI), region-based, for T (n = 59) and for N (n = 217).

Visual Criteria (Region-Based)	T (n = 59)			N (n = 217)		
	FDG	FAPI	*p*-Value	FDG	FAPI	*p*-Value
**TP**	38	45	n.s.	9	8	1
**FN**	15	8		6	7	1
**FP**	3	2		25	7	**0.0018**
**TN**	3	4		177	195	**0.019**
**Sensitivity**	72%	85%		60%	53%	1
**Specificity**	50%	67%		88%	97%	**0.001**
**PPV**	93%	96%		26%	53%	0.135
**NPV**	17%	33%		97%	97%	1
**LR+**	1.43	2.55		4.85	15.39	1
**LR−**	0.57	0.23		0.46	0.48	1
**Accuracy**	69%	83%		86%	94%	**0.018**

Legend: TP = True positive; FN = False negative; FP = False positive; TN = True negative; PPV = positive predictive value; NPV = negative predictive value; LR = likelihood ratio; n.s. = not significant.

**Table 6:** PET/CT diagnostic performance using semiquantitative PET parameters (FDG versus FAPI), region-based, for T (n = 59).

Semiquantitative PET Parameters (Region-Based)	T (n = 59)														
	FDG							FAPI							*p*-Value AUCs (FDGvs. FAPI)
	AUC	95% CI	*p*-Value	Cut-Off	Sensitivity	Specificity	Accuracy	AUC	95% CI	*p*-Value	Cut-Off	Sensitivity	Specificity	Accuracy	Not Significant
**SUVmax-T (Tmax)**	44.2%	0.1322–0.7515	0.325	12	81%	33%	76%	61.2%	0.3342–0.889	0.189	2.1	71%	67%	72%	0.6848
**TBR-L (Tmax/Liver mean)**	55.2%	0.267–0.8368	0.344	0.9	74%	50%	71%	63.7%	0.3347–0.9389	0.140	0.9	89%	50%	84%	0.294
**TBR-MBP (Tmax/MBP mean)**	52.8%	0.236–0.8206	0.415	1	76%	50%	73%	65.1%	0.3573–0.9445	0.116	2.3	77%	67%	76%	0.1147
**TBR-P (Tmax/Lung mean)**	57.5%	0.2656–0.8853	0.277	4	81%	50%	78%	63.2%	0.3226–0.9415	0.148	6.5	72%	67%	71%	0.4142

Legend: SUV = standardised uptake value; TBR = target to background ratio; MBP = mediastinal blood pool; P = pulmonary = lung; AUC = area under the curve; CI = confidence interval.

**Table 7:** PET/CT diagnostic performance using semiquantitative PET parameters (FDG vs. FAPI), region-based, for N (n=217).

Semiquantitative PET Parameters (Region-Based)	N (n = 217)														
	FDG							FAPI							*p*-Value AUCs (FDGsFAPI)
	AUC	95% CI	*p*-Value	Cut-Off	Sensitivity	Specificity	Accuracy	AUC	95% CI	*p*-Value	Cut-Off	Sensitivity	Specificity	Accuracy	Not Significant
**SUVmax-N (Nmax)**	74.7%	0.5968–0.8962	**<0.001**	3	60%	90%	87%	79.9%	0.6477–0.9094	**<0.001**	1.5	73%	83%	82%	0.4314
**TBR-L (Nmax/Liver mean)**	74.7%	0.6133–0.8797	**<0.001**	1.4	60%	91%	88%	78.2%	0.6538–0.9099	**<0.001**	1.1	60%	96%	94%	0.4577
**TBR-MBP (Nmax/MBP mean)**	74.4%	0.6098–0.8783	**<0.001**	1.7	60%	90%	88%	77.9%	0.6477–0.9094	**<0.001**	1.7	60%	96%	94%	0.4642
**TBR-P (Nmax/Lung mean)**	74.9%	0.6127–0.8846	**<0.001**	5.4	60%	90%	88%	77.7%	0.6465–0.9071	**<0.001**	3.2	60%	96%	93%	0.5757

Legend: SUV = standardised uptake value; TBR = target to background ratio; MBP = mediastinal blood pool; P = pulmonary lung; AUC = area under the curve; CI = confidence interval.

## Data Availability

Data is available on request due to privacy restrictions.

## Supplementary Materials

The following supporting information can be downloaded at: https://www.mdpi.com/article/10.3390/ph17111468/s1, Table S1: FDG and FAPI uptake of reference backgrounds within the surgical cohort (n = 50).
